# Evidence for the Effectiveness of Health and Social Services Partnerships

**DOI:** 10.1093/geroni/igaa057.727

**Published:** 2020-12-16

**Authors:** Traci Wilson, Suzanne Kunkel, Amanda Brewster, Jane Straker, Elizabeth Blair, Marisa Scala-Foley

**Affiliations:** 1 Miami University, Oxford, Ohio, United States; 2 University of California, Berkeley, Berkeley, California, United States; 3 National Association of Area Agencies on Aging, Washington, District of Columbia, United States

## Abstract

Integration of health and social services is touted as a key method to address social needs and improve population health. We will share the latest evidence on how Area Agency on Aging (AAA) partnerships with health care entities and other organizations improve health outcomes for older adults, while reducing health care costs. AAAs are community leaders in cross-sectoral partnerships that effectively address social determinants of health for older adults, who account for a substantial share of overall health care spending. Results of a longitudinal study (2008 – 2016) which links data from four waves of the National Surveys of AAAs to data on county-level health outcomes show that AAA–health care partnerships and programs reduced health care utilization and costs. AAA partnerships with hospitals reduced Medicare spending by $136 per beneficiary. AAA involvement in evidence-based health promotion programs decreased potentially avoidable nursing home use by nearly one percentage point (representing a change of 6.5%). Finally, we will describe the prevalence and nature of contracting relationships between community-based organizations and health care entities, based on data from the 2020 CBOs and Health Care Contracting Request for Information, the third national RFI of AAAs, Centers for Independent Living, and other aging and disability community-based organizations.

